# Motivation and Treatment Credibility Predicts Dropout, Treatment Adherence, and Clinical Outcomes in an Internet-Based Cognitive Behavioral Relaxation Program: A Randomized Controlled Trial

**DOI:** 10.2196/jmir.5352

**Published:** 2016-03-08

**Authors:** Sven Alfonsson, Erik Olsson, Timo Hursti

**Affiliations:** ^1^ Clinical Psychology in Healthcare Department of Public Health and Caring Sciences Uppsala University Uppsala Sweden; ^2^ Clinical Psychology Department of Psychology Uppsala University Uppsala Sweden

**Keywords:** Internet, adherence, psychotherapy, motivation, patient compliance

## Abstract

**Background:**

In previous research, variables such as age, education, treatment credibility, and therapeutic alliance have shown to affect patients’ treatment adherence and outcome in Internet-based psychotherapy. A more detailed understanding of how such variables are associated with different measures of adherence and clinical outcomes may help in designing more effective online therapy.

**Objective:**

The aims of this study were to investigate demographical, psychological, and treatment-specific variables that could predict dropout, treatment adherence, and treatment outcomes in a study of online relaxation for mild to moderate stress symptoms.

**Methods:**

Participant dropout and attrition as well as data from self-report instruments completed before, during, and after the online relaxation program were analyzed. Multiple linear and logistical regression analyses were conducted to predict early dropout, overall attrition, online treatment progress, number of registered relaxation exercises, posttreatment symptom levels, and reliable improvement.

**Results:**

Dropout was significantly predicted by treatment credibility, whereas overall attrition was associated with reporting a focus on immediate consequences and experiencing a low level of intrinsic motivation for the treatment. Treatment progress was predicted by education level and treatment credibility, whereas number of registered relaxation exercises was associated with experiencing intrinsic motivation for the treatment. Posttreatment stress symptoms were positively predicted by feeling external pressure to participate in the treatment and negatively predicted by treatment credibility. Reporting reliable symptom improvement after treatment was predicted by treatment credibility and therapeutic bond.

**Conclusions:**

This study confirmed that treatment credibility and a good working alliance are factors associated with successful Internet-based psychotherapy. Further, the study showed that measuring adherence in different ways provides somewhat different results, which underscore the importance of carefully defining treatment adherence in psychotherapy research. Lastly, the results suggest that finding the treatment interesting and engaging may help patients carry through with the intervention and complete prescribed assignments, a result that may help guide the design of future interventions.

**Trial Registration:**

Clinicaltrials.gov NCT02535598; http://clinicaltrials.gov/ct2/show/NCT02535598 (Archived by WebCite at http://www.webcitation.org/6fl38ms7y).

## Introduction

It is well established that therapist-guided Internet-based cognitive behavior therapy (ICBT) and other behavioral interventions can be effective in improving psychological symptoms and well-being [1]. However, not all patients are helped and one reason for this could be that treatment adherence may be somewhat lower in Internet-based psychotherapy compared to traditional face-to-face therapy [2]. This difference may be important because adherence is associated with treatment outcome in both face-to-face and Internet-based therapy [3]. Little is known about factors that affect treatment adherence to Internet-based interventions, but it is clear that therapist online support improves adherence substantially [4]. With the growth of Internet-based interventions, there has been an increased interest in trying to find variables that affect treatment adherence and outcomes [5-7]. However, the association between treatment adherence and outcome is complex and in previous studies it has been shown that increased adherence does not automatically lead to better treatment outcomes [8,9]. It is also unknown to what degree treatment adherence can be affected by therapist behaviors because background variables such as education and personality traits affect patients’ ability to carry through and benefit from ICBT [10]. In order to develop and evaluate effective Internet-based interventions, it is important to further investigate factors that are associated with adherence and outcome [11,12].

Adherence to Internet-based treatments can be operationalized in different ways, including working with the intervention by reading texts and watching video clips on a webpage or adhering to the behavioral prescriptions in everyday life (eg, by completing homework assignments) [6,13]. In other words, one may separate the administration of the intervention (ie, providing the treatment content) and the proposed behavioral mechanisms (ie, everyday behavior change) that more directly lead to symptom change [14-16]. In Internet interventions, therapist support is hypothesized to help participants both to work with the online material and to complete behavioral assignments. The effect of human support can be explained by operant principles and by models such as self-determination theory (SDT), which is a framework that describes motivation and behavior change in congruence with operant conditioning principles [17]. According to SDT, behavior is governed by external motivation (behavior governed by external factors or consequences) and four forms of internal motivation: introjected motivation (behavior governed by reduced negative affect), identified and integrated motivation (behavior governed by goals or values), and intrinsic motivation (behavior that is in itself rewarding).

Self-determination theory has been applied to health behaviors and it seems that different forms of motivation may have different effects on treatment adherence [18]. For example, behaviors that are governed by intrinsic, identified, or integrated motivation are more likely to occur and to be enduring over time compared to behaviors that are governed by external motivation. Because some behavioral prescriptions in psychotherapy, such as exposure exercises, are not intrinsically rewarding, patients are helped by therapists to instead employ identified or integrated motivation to facilitate behavior change [19]. That therapists’ follow-up on assignments may also be an example where introjected or external motivation may be beneficiary for treatment adherence.

Previous studies have shown that adherence to an intervention, as well as treatment outcome, may be influenced by demographical variables, such as gender, age, and education [20-22]. Another background factor that may be important in this context is the ability to focus on either future or present consequences [23-25]. According to Zimbardo’s model of behavior and postponed reward, there are three types of time perspectives: future, hedonistic, and fatalistic. A future-oriented time perspective is goal-driven and has been associated with health behaviors, whereas the opposite is true for people with the hedonistic time perspective who are highly influenced by immediate consequences and stimulation [26]. Lastly, a person with a fatalistic time perspective may show a pattern of health-destructive behaviors [27]. Thus, time perspective is closely associated with executive functioning and the ability to postpone reinforcement and to act based on future goals rather than present needs [28-30]. Therefore, it may be an important construct that can help explain some of the variance in participants’ adherence to psychological interventions.

Finally, patients’ belief in a treatment may have a very strong effect on the outcome as seen in studies on placebo effects [31]. However, the credibility of a treatment may also explain why a patient engages in treatment and completes assignments that are not intrinsically rewarding. Thus, belief may be associated with internal motivation and can have an indirect but real effect on outcome in the case of psychotherapy [22,32].

This study aimed at investigating variables that may predict three different types of outcome variables in ICBT: (1) dropout and attrition from treatment, (2) treatment adherence, and (3) clinical outcomes. More specifically, the aim was to assess the predictive value of different background variables as well as the variables time perspective, treatment credibility, motivation, and therapeutic bond on early dropout, attrition, treatment progress, adherence to behavioral prescriptions, posttreatment symptoms, and reliable improvement in a previously conducted study of a brief online stress management treatment. A secondary aim was to investigate whether treatment adherence could predict clinical outcomes.

## Methods

### Design, Procedure, and Participants

Data for this study were retrieved from a previously conducted study on Internet-based relaxation training for people with mild to moderate stress and anxiety symptoms [9]. Participants were recruited from the general population primarily by online advertisement. Inclusion criterion was self-reported stress symptoms, whereas the exclusion criteria were elevated symptom levels of anxiety or depression or other severe psychological or somatic problems that warranted immediate care, younger than 18 years of age, insufficient mastery of the Swedish language, or lacking daily access to computer, Internet, and cell phone. In the study, a total of 162 included participants were presented with an online intervention and were randomized to either normal or enhanced treatment presentation and either normal or enhanced therapist support in a full factorial design [9]. In the normal treatment condition, the treatment was presented as black-and-white text files, whereas in the enhanced presentation condition, the treatment was presented in full color and also in video format. In the normal support condition, participants received support from trained therapists at least once a week, whereas in the enhanced support condition, participants received daily support from therapists based on motivational interviewing. The intervention consisted of a brief 4-week program of applied relaxation similar to what has been used and empirically tested in previous studies [33]. Each week of the program included webpage material, assigned relaxation exercises, and contact with a therapist via the webpage. All participants were asked to answer a battery of self-report instruments before, during, and after the intervention; of the 162 participants, 157 had complete data from the pre- and midtreatment assessments, whereas 96 participants had complete data also from the posttreatment assessment.

### Outcome Variables

Early dropout (yes/no) was assessed by counting the number of participants who discontinued the study before completing the first week of the treatment program; attrition (yes/no) was assessed by counting the total number of participants who discontinued the study before the posttreatment assessment. Treatment adherence was divided into the two variables treatment progress and registered exercises, both on continuous scales. Treatment progress was assessed by measuring how much of the Web-based treatment material (eg, texts, examples, assignments) each participant accessed before dropping out or completing the treatment. Because the treatment consisted of 25 such items, this variable ranged from zero (not accessed the treatment) to 25 (accessed the whole treatment). Registered exercises were measured by the mean number of prescribed exercises of applied relaxation that the participant had registered on the webpage each week. The weekly number of registered exercises ranged from zero (not completed any exercises) to 14 (completed all prescribed exercises). As a measure of stress symptoms and treatment outcome, the Perceived Stress Scale (PSS) [34] was chosen. The short version of the PSS used in this study contains 14 items scored on a scale between zero and 4, which provides a total score between zero and 56 with a higher score indicating more symptoms of stress. In the current study, the PSS had an internal reliability of α=.72. In previous studies, the PSS has showed adequate psychometric properties [35]. To assess reliable improvement (yes/no), the Reliable Change Index for PSS was calculated (described subsequently).

### Predictor Variables

The predictor variables consisted of the background variables age, gender, level of education (primary/secondary/university), occupation (student/unemployed/employed/sick leave/retired), and computer expertise (low/intermediate/high). In order to facilitate interpretation, education and occupation were transformed into dichotomous variables (nonuniversity vs university; employed/student/retired vs unemployed/sick leave). Four psychological predictor variables were collected by self-report instruments at baseline: time perspective, treatment credibility, and internal and external motivation. Intrinsic motivation and therapeutic bond were measured at midtreatment and stress symptoms were measured both at baseline as a predictor variable and at postmeasurement as an outcome variable. Because a previous analysis [9] had shown that enhanced support was associated with higher adherence to the online treatment program, treatment condition (normal vs enhanced therapeutic support) was also included as a predictor variable in the analyses. No other independent variable was significantly associated with any outcome variables.

Time perspective was measured with the Zimbardo Time Perspective Inventory Short Form (ZTPI) [36,37]. The version of the ZTPI used in this study has three subscales—future, hedonistic, and fatalistic—and comprise 22 items scored on a scale from 1 to 5 [38]. The ZTPI has been evaluated for research in health psychology and has shown adequate psychometric properties [39]. The ZTPI subscales had internal reliabilities of α=.69-.73 in this study.

Treatment credibility was measured with the Treatment Credibility Scale (TCS), which is often used in studies of Internet interventions and is an adaptation from Borkovec and Nau [40]. The TCS consists of five items scored on a scale from 1 to 10 with a higher score indicating more trust in the current treatment. The TCS had an internal reliability of α=.83 in this study.

Internal (ie, identified and integrated motivation) and external motivation were measured with the Treatment Self-Regulation Questionnaire (TSRQ) [41]. The TSRQ consists of two subscales—internal motivation (IM) and external motivation (EM)—each measured with six items that were adapted to suit the Internet intervention used in this study. Each subscale provides a score between 6 and 42 with a higher score corresponding to a higher degree of motivation. The TSRQ has been used in studies on motivation and health behaviors and has shown adequate psychometric properties [42]. In the present study, the TSRQ-IM and TSRQ-EM had internal reliabilities of α=.70 and α=.68, respectively.

Intrinsic motivation was measured with the Intrinsic Motivation Inventory (IMI) [43]. The IMI aims at measuring how pleasant, interesting, and meaningful a task is perceived and has nine items scored on a scale between 1 and 7 which provides a total score of 9 to 63 with a higher score indicating a more positive experience of the task. Due to mixed findings concerning the factor structure of the IMI, only the total score was used [44]. The IMI had α=.71 in this study.

Therapist bond was measured with the Working Alliance Questionnaire Short Form (WAI) [45]. The WAI has been widely used in psychotherapy research and has shown adequate psychometric properties [46]. The short form WAI consists of three subscales—Goal, Task, and Bond—each with a score between 4 and 28 where a higher score equals a higher degree of therapeutic alliance. The WAI total scale had an internal reliability of α=.73 in this study.

### Analysis

Before analysis, data were screened for outliers and normality, linearity, and homoscedasticity were evaluated by scrutinizing the residual scatterplots between predicted variables and errors of prediction and found adequate. Because subscales were entered into the analyses, multicollinearity was assessed by analyzing the variance inflation factor for each predictor variable and found to be nonproblematic.

Reliable improvement was computed by dividing the difference between the pretreatment and posttreatment scores by the standard error of the difference between the two scores. If the Reliable Change Index was greater than 1.96, a change of that magnitude would not be expected due to the unreliability of the measure [47]. Using this procedure, the reliable improvement criterion for PSS was a change score of 10 or more in this study.

Modeled after deGraaf et al [48], bivariate regression analysis was first used to identify candidate (*P*<.10) predictor variables for each outcome variable. All identified predictor variables were included in subsequent multiple regression analyses using a backward deletion process for each outcome variable. Logistic regression was used for the dichotomous outcome variables dropout and attrition and linear regression was used for continuous outcome variables. Cox-Snell *R* ^2^ and Nagelkerke *R* ^2^ were used as a measure of overall model fit in the logistic regression analyses and *R* ^2^ was used in the linear regression analyses. Because some of the variables had distributions that deviated somewhat from normality, the final regression models were confirmed using robust regression analyses with bootstrap and bias correction. The sample size of 157 was deemed adequate for regression analysis of a maximum of eight predictor variables for each outcome variable except stress symptoms and reliable improvement for which the sample size of 96 was deemed adequate for six predictor variables. Single missing values (n<1%) were imputed using expectation-maximization estimates. A *P* value of .05 was considered the threshold for statistical significance if not stated otherwise; exact *P* values were reported for the final analyses.

## Results

Of the 157 participants in this study, 115 (73.2%) were women and the mean age was 34.5 (SD 13.1) years. The background variables education, occupation, and computer expertise are shown in Table 1. A total of 39 of 157 (24.8%) participants dropped out before completing the first week of treatment; the total attrition was 61 (38.9%) participants at the postmeasurement. The mean treatment progress among all participants was 14.9 (SD 9.7) items out of 25 (60%) and the mean number of weekly exercises was 8.5 (SD 4.0) out of the prescribed 14 (61%).

**Table 1:** Background predictor variables (N=157).

Factor		n (%)
**Education**		
	Primary	8 (5.1)
	Secondary	57 (36.3)
	University	92 (58.6)
**Occupation**		
	Studying	43 (27.4)
	Unemployed	9 (5.7)
	Employed	81 (51.6)
	Sick leave	16 (10.2)
	Retired	8 (5.1)
**Computer expertise**		
	Low	54 (34.4)
	Intermediate	51 (32.5)
	High	52 (33.1)

There were no significant differences for any of the predictor variables between the treatment groups of the original study. Of the background variables, only education and occupation were significant predictors for any outcome variable in the initial bivariate regression analyses. Of the self-reported psychological variables, the TSRQ-IM showed markedly higher standard deviation compared to other variables and it was the only variable that failed to significantly predict any outcome variable, so it was removed from further analyses. The results of these bivariate analyses for each outcome variable can be found in Multimedia Appendices 1 and 2.

### Dropout and Attrition

The multivariate logistic regression analyses showed that early dropout could be significantly negatively predicted by the TCS (B=–0.14, χ^2^_1_=10.5, *P*=.001), whereas total attrition to postmeasurement was predicted by baseline stress symptoms (B=0.08, χ^2^_1_=3.2, *P*=.05), the ZTPI Hedonistic subscale (B=0.32, χ^2^_1_=10.3, *P*=.001), and the IMI (B=–0.06, χ^2^_1_=5.7, *P*=.02) (see Table 2). Early dropout was associated with a low belief in the treatment model, whereas dropout during the course of treatment was associated with having elevated stress symptoms, being more focused on the immediate consequences of behaviors, and finding the treatment uninteresting or unengaging.

**Table 2:** Significant predictor variables for early dropout and attrition after backward deletion (N=157).

Treatment dropout^a^		Cox-Snell *R* ^ 2 ^	Nagelkerke *R* ^ 2 ^	B (SE)	χ^2^_1_	*P*	OR (95% CI)
**Early dropout**		.18	.39				
	TCS			–0.14 (0.04)	10.5	.001	0.87 (0.80-0.95)
**Attrition**		.19	.28				
	Baseline stress symptoms			0.08 (0.05)	3.2	.05	1.08 (1.00-1.18)
	ZTPI Hedonistic			0.32 (0.10)	10.3	.001	1.37 (1.13-1.66)
	IMI			–0.06 (0.03)	5.7	.02	0.94 (0.90-0.99)

^a^IMI: Intrinsic Motivation Inventory; TCS: Treatment Credibility Scale; ZTPI: Zimbardo Time Perspective Inventory.

### Treatment Adherence

After controlling for level of support, treatment progress was positively predicted by level of education (beta=.24, *t*_153_=2.33, *P*=.02) and by the TCS (beta=.35, *t*_153_=3.36, *P*=.001), whereas registered exercises was significantly predicted only by the IMI (beta=.29, *t*_155_=2.43, *P*=.02) (see Table 3). All other predictor variables were nonsignificant in the multiple regression analyses for treatment adherence. Accessing the online treatment material was associated with a priori belief in the treatment model, whereas complying with the prescribed homework assignments was associated with experiencing interest and engagement in the treatment.

**Table 3:** Significant predictor variables for treatment adherence after backward deletion (N=157).

Treatment adherence^a^		*R* ^ 2 ^	B (SE)	β	*t* (*df*)	*P*
**Treatment progress**		.36				
	Enhanced support		2.84 (0.88)	.33	3.23 (153)	.002
	University education		1.95 (0.84)	.24	2.33 (153)	.02
	TCS		0.15 (0.04)	.35	3.36 (153)	.001
**Registered exercises**		.13				
	IMI		0.10 (0.04)	.29	2.43 (155)	.02

^a^IMI: Intrinsic Motivation Inventory; TCS: Treatment Credibility Scale.

### Treatment Outcome

Posttreatment stress symptoms were significantly and positively predicted by the baseline stress symptoms (beta=.47, *t*_91_=4.43, *P*<.001) and by the TSRQ-EM (beta=.25, *t*_91_=2.40, *P*=.02) while negatively predicted by the TCS (beta=–.28, *t*_91_=3.53, *P*=.001) (see Table 4). Reliable improvement was positively predicted by baseline stress symptoms (B=0.11, χ^2^_1_=4.5, *P*=.03), by the TCS (B=0.09, χ^2^_1_=3.3, *P*=.05) and by the WAI (B=0.14, χ^2^_1_=3.9, *P*=.049) (see Table 5). Reporting external pressure to complete the treatment was associated with worse treatment outcome, whereas a good therapeutic bond was associated with a substantial positive treatment effect. Treatment credibility predicted both overall symptom levels and substantial improvement.

**Table 4:** Significant predictor variables for post treatment stress symptoms after stepwise deletion (n=96).

Predictor variable^a^	*R* ^ 2 ^	B (SE)	β	*t*_91_	*P*
Postmeasurement stress symptoms	.42				
Baseline stress symptoms		0.53 (0.12)	.47	4.43	<.001
TSRQ-EM		0.48 (0.20)	.25	2.40	.02
TCS		–0.28 (0.08)	–.35	3.53	.001

^a^TCS: Treatment Credibility Scale; TSRQ-EM: Treatment Self-Regulation Questionnaire Extrinsic Motivation.

**Table 5:** Significant predictor variables for reliable improvement after stepwise deletion (n=96).

Predictor variable^a^	Cox-Snell *R* ^ 2 ^	Nagelkerke *R* ^ 2 ^	B (SE)	χ^2^_1_	*P*	OR (95% CI)
Reliably improved	.32	.47				
Baseline stress symptoms			0.11 (0.05)	4.5	.03	1.12 (1.01-1.25)
TCS			0.09 (0.05)	3.3	.05	1.10 (1.00-1.20)
WAI total			0.14 (0.07)	3.9	.049	1.15 (1.01-1.32)

^a^TCS: Treatment Credibility Scale; WAI: Working Alliance Inventory.

### Associations Between Treatment Adherence and Outcomes

In bivariate regression analyses and after controlling for baseline PSS score, posttreatment stress symptoms was significantly negatively predicted by both treatment progress (beta=–.31, *t*_92_=3.29, *P*=.001) and registered exercises (beta=–.21, *t*_92_=1.98, *P*=.05). The same pattern was seen for reliable improvement, which was significantly predicted by both treatment progress (B=0.26, χ^2^_1_=8.0, *P*=.005; OR 1.29, 95% CI 1.08-1.55) and registered exercises (B=0.20, χ^2^_1_=7.5, *P*=.006; OR 1.23, 95% CI 1.06-1.42). Whether treatment effect was mediated through treatment adherence could not be further investigated in this study because the power calculations had not accounted for this type of analyses.

## Discussion

### Predicting Dropout, Attrition, and Adherence

The analyses showed that different ways of operationalizing (ie, measuring) dropout, treatment adherence, and treatment outcomes was related to somewhat different predictor variables, something that may be important to consider in psychotherapy process research. First, there was a difference in that early treatment dropout could be predicted by treatment credibility, whereas further attrition was predicted by a personality pattern of focusing of immediate consequences and by finding the treatment unengaging, two variables that are probably connected. People whose behavior is generally governed by immediate consequences and reward may find an online behavioral treatment largely consisting of abstract instructions and texts unsatisfying and boring. This pattern suggests that some people with low expectations of the treatment start the program, but very soon realize it does not suit them and quit. Whether these participants should receive more motivational support from therapists to stay in the online treatment or efforts should be made to guide them to other forms of treatment needs further investigation. Participants who dropped out later in the treatment reported a lower tendency to focus on future goals and also experienced the treatment as unrewarding. These results are in line with previous results showing that being able to focus on the future and to postpone rewards is associated with higher levels of education and health behaviors [23]. Although the treatment may be designed to be more interesting and engaging (eg, by employing media content or using features of gamification [49]), it may be difficult to counter the fact that most health behaviors rely on an ability to postpone rewards and a focus on future consequences. However, strategies such as repeated reminding of treatment goals and using everyday prompts may be alternatives for further investigation [50].

Treatment adherence showed a somewhat similar pattern to dropout with online treatment progress predicted by education and treatment credibility. Thus, working with the online material was associated with a probable familiarity of working with texts and thinking in abstract terms. Treatment credibility may correspond to a familiarity and interest in using the Internet for learning about health behaviors and perhaps previous positive experiences of online courses. Treatment credibility may also represent participants’ belief in the treatment model and specifically in using relaxation to target stress symptoms. Finally, the association between treatment credibility and treatment progress may represent the different reasons why people undergo Internet-based psychotherapy; for some it is a preferred choice, whereas for others it may be the only available option. Completion of homework assignments, similar to staying in the treatment program, was predicted by finding the treatment engaging. That the IMI was significantly associated with adherence in this study may be partly explained by the nature of relaxation exercises that may actually be pleasant in contrast to exposure exercises, for example. It is worth noting that the instrument used to measure intrinsic motivation, the IMI, is designed to measure different forms of intrinsic reward and comprise items concerning experiences of interest, enjoyment, and meaningfulness [41]. It may capture participants’ experience that the treatment suits them and their needs rather than the treatment being “fun.” Whether results from this study of relaxation exercises can be generalized to other types of assignments used in CBT needs further study. In short, the results from this study suggest that a high level of education and belief in the treatment model may be beneficiary for overcoming the effort needed to work with the online program, but participants who find the treatment suits them work with the prescribed assignments to a higher degree. However, few of the variables in this study could significantly predict adherence to the assignments, so there are probably other unknown variables that could help explain this behavior.

### Predicting Treatment Outcomes

Among participants who did not dropout and remained in the study, treatment outcome, as measured by the posttreatment stress symptoms, was significantly positively predicted by baseline stress symptoms, but also by the TSRQ-EM measuring external motivation. External motivation corresponds to feeling pressured by others, mostly in a negative way, to complete tasks. In previous studies, external motivation has been associated with difficulties in sustaining behavior and a reliance on accountability [19]. That external motivation was associated with worse outcome in this study implies that people who complete the intervention because of feeling obliged to do so benefit less from the treatment. In a recent study of negative effects in online psychotherapy, some participants reported feeling pressured by their online therapist or the demands of the treatment schedule [51], but in this study, external motivation was measured at baseline and corresponded to the reasons why participants took part in the study. Why some participants reported external pressure is unknown and difficult to speculate about because this study was based on self-referral recruitment. It is important to note the different nature of psychotherapy compared to the workplace environment, where most of the research on motivation has been conducted. In studies of psychotherapy, results may rely more on participants being engaged and involved than in most workplace research. For example, unlike work tasks, psychotherapy aims at making participants generalize behaviors from the therapy situation to their everyday life, something that may be more probable if the patient experiences the treatment as meaningful. Also, the Internet format may deemphasize external motivation because of the perceived anonymity of both patients and participants [52,53]. Previous studies have shown that therapists’ prompts and reminders can have a relatively minor impact on treatment adherence among participants who may lack internal motivation [54]. Posttreatment stress symptoms were further negatively predicted by treatment credibility, an effect also seen in previous studies [21,22]. Belief in the treatment format and model seems to be a consistent predictor for positive outcome and may be used as a measure for identifying people for whom the online format and minimal therapist contact may be beneficiary. It is important to note that suggesting or persuading people to try Internet-based psychotherapy or a treatment model they do not believe in may be counterproductive, both given the effect seen for external motivation and the effect of treatment credibility on treatment outcome. However, given the positive results of many online behavioral interventions, it may be beneficiary to better inform skeptical participants about the treatment format (eg, by pretreatment vignettes or examples). After controlling for baseline stress symptoms, reliable improvement after the treatment program was predicted by treatment credibility, but also by therapeutic bond, an effect that has been seen in other studies as well [55]. Interestingly, this effect in this study was seen only among participants who improved substantially, further providing support for the notion that working alliance is as an important variable in online psychotherapy as in face-to-face therapy [56].

### Identified Difficulties and Study Limitations

In this study, many of the proposed predictor variables could not significantly predict the outcome variables in the multivariate analyses. This suggests that several of the predictor variables covaried to a large degree; therefore, finding the best predictor variables is difficult. The multivariate analyses with backward deletion resulted in models with acceptable levels of model fit except for registered exercises (*R* ^2^=.13); so far, there is limited understanding of the processes that may be involved in following prescribed homework in ICBT. That personality traits associated with conscientiousness and delay discounting could predict carrying through with the treatment seemed reasonable, but it was somewhat surprising that working alliance could not. A strong working alliance is often associated with treatment adherence, but in this study this association was overshadowed by the impact of education and treatment credibility. The weak association between working alliance and treatment adherence was unexpected given that therapeutic alliance has shown to be strongly associated with adherence and outcome in several other studies of ICBT [57]. Finding the intervention engaging, interesting, and meaningful were crucial factors for participants in successful ICBT as seen in previous studies [58]. Although intrinsic motivation was a predictor for treatment adherence, it was not associated with treatment outcome; this was surprising given the association between treatment adherence and outcome. This study was not designed to investigate mediation effect; therefore; it was unfortunately underpowered to further analyze these processes.

This study has a number of limitations. First, several of the predictor and outcome variables were difficult to measure accurately. For example, the measurement of registered exercises was constructed to assess the treatment’s effective mechanism, but may have failed to fully capture changes in participants’ everyday behavior. The intervention encouraged participants to conduct relaxation exercises and highly engaged participants may have done so without registering on the webpage. Further, several of the self-report instruments have not been used in psychotherapy research before and their psychometric properties in this context are unknown. For example, the internal reliability of some questionnaires seemed to be somewhat lower in this study compared to previously reported figures. Second, a more complex design with repeated measurements of adherence could have been conducted to show causal mediation, but this would also have demanded a much larger sample size. Third, the lack of a control group or a face-to-face treatment condition limits the generalizability of the results. Therefore, whether the conclusions from this study are valid for other treatment modalities are unknown. Finally, and most importantly, there was large dropout (39%) at postmeasurement and although investigating dropout from Internet-based interventions was one of the aims of this study, it also meant loss of follow-up data. This also means that the conclusions regarding predictors of clinical outcome are only valid for participants who stay in the study or treatment, which limits the generalizability of the results. A more thorough analysis of participants who dropped out may have resulted in a better understanding of this group.

### Conclusions

In conclusion, this study confirms the importance of treatment credibility and working alliance in Internet-based psychotherapy and also suggests that experiencing intrinsic reward from participating in the treatment may be important. In contrast, external pressure to try online therapy may be counterproductive and lead to worse outcomes. Apart from identifying people who believe in the online treatment format and continue to explore the best methods for online therapist support, it may also be valuable to further investigate what makes participants engage in an intervention and what features makes the intervention interesting and meaningful [55]. However, given that quite rudimentary online treatment programs seem to be effective, it is possible that Internet-based interventions simply suit some participants better than others and that the focus should be on identifying these participants early [59]. Future studies may investigate both what makes treatments engaging while recognizing that different participants may have different requests and wishes for an intervention [60]. A good match between participant expectations and needs on the one hand and the intervention content and design on the other hand may be one of the reasons clinical assessment before treatment and tailoring treatment to better suit different patients may be beneficiary for treatment outcomes in Internet-based interventions [61]. Believing in the online treatment format and finding working with the online program rewarding may be two aspects of a process that could be investigated further to develop even more effective treatment programs.

## Appendix

Multimedia Appendix 1 Identified candidate predictor variables from the bivariate analyses for nominal outcome variables.

Multimedia Appendix 2 Identified candidate predictor variables from the bivariate analyses for continous outcome variables.

## Abbreviations

ICBT: Internet-based cognitive behavior therapy
IM: internal motivation
IMI: Intrinsic Motivation Inventory
PSS: Perceived Stress Scale
SDT: self-determination theory
TCS: Treatment Credibility Scale
TSRQ: Treatment Self-Regulation Questionnaire

## References

[1] Spek V, Cuijpers P, Nyklícek I, Riper H, Keyzer J, Pop V (2007). Internet-based cognitive behaviour therapy for symptoms of depression and anxiety: a meta-analysis. Psychol Med.

[2] van Ballegooijen W, Cuijpers P, van Straten A, Karyotaki E, Andersson G, Smit JH, Riper H (2014). Adherence to Internet-based and face-to-face cognitive behavioural therapy for depression: a meta-analysis. PLoS One.

[3] Mausbach BT, Moore R, Roesch S, Cardenas V, Patterson TL (2010). The relationship between homework compliance and therapy outcomes: an updated meta-analysis. Cognit Ther Res.

[4] Hilvert-Bruce Z, Rossouw PJ, Wong N, Sunderland M, Andrews G (2012). Adherence as a determinant of effectiveness of internet cognitive behavioural therapy for anxiety and depressive disorders. Behav Res Ther.

[5] Kelders SM, Kok RN, Ossebaard HC, Van Gemert-Pijnen JE (2012). Persuasive system design does matter: a systematic review of adherence to web-based interventions. J Med Internet Res.

[6] Donkin L, Christensen H, Naismith SL, Neal B, Hickie IB, Glozier N (2011). A systematic review of the impact of adherence on the effectiveness of e-therapies. J Med Internet Res.

[7] Høifødt RS, Mittner M, Lillevoll K, Katla SK, Kolstrup N, Eisemann M, Friborg O, Waterloo K (2015). Predictors of response to Web-based cognitive behavioral therapy with high-intensity face-to-face therapist guidance for depression: a Bayesian analysis. J Med Internet Res.

[8] Titov N, Dear BF, Johnston L, McEvoy PM, Wootton B, Terides MD, Gandy M, Fogliati V, Kayrouz R, Rapee RM (2014). Improving adherence and clinical outcomes in self-guided internet treatment for anxiety and depression: a 12-month follow-up of a randomised controlled trial. PLoS One.

[9] Alfonsson S, Olsson E, Hursti T (2015). The effects of therapist support and treatment presentation on the clinical outcomes of an Internet based applied relaxation program. Internet Interventions.

[10] Button KS, Wiles NJ, Lewis G, Peters TJ, Kessler D (2012). Factors associated with differential response to online cognitive behavioural therapy. Soc Psychiatry Psychiatr Epidemiol.

[11] Ritterband LM, Thorndike FP, Cox DJ, Kovatchev BP, Gonder-Frederick LA (2009). A behavior change model for internet interventions. Ann Behav Med.

[12] Mohr DC, Cuijpers P, Lehman K (2011). Supportive accountability: a model for providing human support to enhance adherence to eHealth interventions. J Med Internet Res.

[13] Eysenbach G (2005). The law of attrition. J Med Internet Res.

[14] Kazdin AE (2007). Mediators and mechanisms of change in psychotherapy research. Annu Rev Clin Psychol.

[15] Dunn G, Maracy M, Dowrick C, Ayuso-Mateos JL, Dalgard OS, Page H, Lehtinen V, Casey P, Wilkinson C, Vazquez-Barquero JL, Wilkinson G (2003). Estimating psychological treatment effects from a randomised controlled trial with both non-compliance and loss to follow-up. Br J Psychiatry.

[16] Donkin L, Hickie IB, Christensen H, Naismith SL, Neal B, Cockayne NL, Glozier N (2013). Rethinking the dose-response relationship between usage and outcome in an online intervention for depression: randomized controlled trial. J Med Internet Res.

[17] Deci EL, Ryan RM, Van Lange P, Kruglanski A, Higgins T (2011). Self-determination theory. Handbook of Theories of Social Psychology.

[18] Ryan RM, Patrick H, Deci EL, Williams GC (2008). Facilitating health behaviour change and its maintenance: Interventions based on self-determination theory. Eur Health Psychologist.

[19] Ryan RM, Deci EL (2008). A self-determination theory approach to psychotherapy: the motivational basis for effective change. Can Psychol.

[20] Melville KM, Casey LM, Kavanagh DJ (2010). Dropout from Internet-based treatment for psychological disorders. Br J Clin Psychol.

[21] El Alaoui S, Ljótsson B, Hedman E, Kaldo V, Andersson E, Rück C, Andersson G, Lindefors N (2015). Predictors of symptomatic change and adherence in Internet-based cognitive behaviour therapy for social anxiety disorder in routine psychiatric care. PLoS One.

[22] Karyotaki E, Kleiboer A, Smit F, Turner DT, Pastor AM, Andersson G, Berger T, Botella C, Breton JM, Carlbring P, Christensen H, de Graaf E, Griffiths K, Donker T, Farrer L, Huibers MJ, Lenndin J, Mackinnon A, Meyer B, Moritz S, Riper H, Spek V, Vernmark K, Cuijpers P (2015). Predictors of treatment dropout in self-guided web-based interventions for depression: an 'individual patient data' meta-analysis. Psychol Med.

[23] Boyd J, Zimbardo P, Strathman A, Joireman J (2005). Time perspective, health, and risk taking. Understanding Behavior in the Context of Time: Theory, Research, and Application.

[24] Daugherty JR, Brase GL (2010). Taking time to be healthy: predicting health behaviors with delay discounting and time perspective. Pers Indiv Differ.

[25] Vangberg HCB, Lillevoll KR, Waterloo K, Eisemann M (2012). Does personality predict depression and use of an Internet-based intervention for depression among adolescents?. Depress Res Treat.

[26] Wills TA, Sandy JM, Yaeger AM (2001). Time perspective and early-onset substance use: a model based on stress-coping theory. Psychol Addict Behav.

[27] Henson JM, Carey MP, Carey KB, Maisto SA (2006). Associations among health behaviors and time perspective in young adults: model testing with boot-strapping replication. J Behav Med.

[28] Jurado MB, Rosselli M (2007). The elusive nature of executive functions: a review of our current understanding. Neuropsychol Rev.

[29] Wittmann M, Paulus MP (2008). Decision making, impulsivity and time perception. Trends Cogn Sci.

[30] Teuscher U, Mitchell S (2011). The Psychological Record.

[31] Kaptchuk TJ, Friedlander E, Kelley JM, Sanchez MN, Kokkotou E, Singer JP, Kowalczykowski M, Miller FG, Kirsch I, Lembo AJ (2010). Placebos without deception: a randomized controlled trial in irritable bowel syndrome. PLoS One.

[32] McQueen D, Cohen S, St John-Smith P, Rampes H (2013). Rethinking placebo in psychiatry: how and why placebo effects occur. Adv Psychiat T.

[33] Carlbring P, Björnstjerna E, Bergström AF, Waara J, Andersson G (2007). Applied relaxation: an experimental analogue study of therapist vs. computer administration. Comput Hum Behav.

[34] Cohen S, Kamarck T, Mermelstein R (1983). A global measure of perceived stress. J Health Soc Behav.

[35] Lee E (2012). Review of the psychometric evidence of the perceived stress scale. Asian Nurs Res (Korean Soc Nurs Sci).

[36] Carelli M, Wiberg B, Wiberg M (2011). Development and construct validation of the Swedish Zimbardo Time Perspective Inventory. Eur J Psychol Assess.

[37] Zimbardo P, Boyd J (1999). Putting time in perspective: a valid, reliable individual-differences metric. J Pers Soc Psychol.

[38] D’alessio M, Guarino A, De Pascalis V, Zimbardo P (2003). Testing Zimbardo's Stanford Time Perspective Inventory (STPI)-short form an Italian study. Time Soc.

[39] Crockett RA, Weinman J, Hankins M, Marteau T (2009). Time orientation and health-related behaviour: measurement in general population samples. Psychol Health.

[40] Borkovec T, Nau S (1972). Credibility of analogue therapy rationales. J Behav Ther Exp Psy.

[41] Deci E, Ryan R (1985). Intrinsic Motivation and Self-Determination in Human Behavior.

[42] Levesque CS, Williams GC, Elliot D, Pickering MA, Bodenhamer B, Finley PJ (2007). Validating the theoretical structure of the Treatment Self-Regulation Questionnaire (TSRQ) across three different health behaviors. Health Educ Res.

[43] McAuley E, Duncan T, Tammen VV (1989). Psychometric properties of the Intrinsic Motivation Inventory in a competitive sport setting: a confirmatory factor analysis. Res Q Exerc Sport.

[44] Markland D, Hardy L (1997). On the factorial and construct validity of the Intrinsic Motivation Inventory: conceptual and operational concerns. Res Q Exerc Sport.

[45] Hatcher R, Gillaspy J (2006). Development and validation of a revised short version of the working alliance inventory. Psychother Res.

[46] Munder T, Wilmers F, Leonhart R, Linster HW, Barth J (2010). Working Alliance Inventory-Short Revised (WAI-SR): psychometric properties in outpatients and inpatients. Clin Psychol Psychother.

[47] Bauer S, Lambert MJ, Nielsen SL (2004). Clinical significance methods: a comparison of statistical techniques. J Pers Assess.

[48] de Graaf LE, Hollon SD, Huibers MJ (2010). Predicting outcome in computerized cognitive behavioral therapy for depression in primary care: A randomized trial. J Consult Clin Psychol.

[49] Cugelman B (2013). Gamification: what it is and why it matters to digital health behavior change developers. JMIR Serious Games.

[50] Clarke G, Eubanks D, Reid E, Kelleher C, O'Connor E, DeBar LL, Lynch F, Nunley S, Gullion C (2005). Overcoming Depression on the Internet (ODIN) (2): a randomized trial of a self-help depression skills program with reminders. J Med Internet Res.

[51] Rozental A, Boettcher J, Andersson G, Schmidt B, Carlbring P (2015). Negative effects of internet interventions: a qualitative content analysis of patients' experiences with treatments delivered online. Cogn Behav Ther.

[52] Zur O, Williams M, Lehavot K, Knapp S (2009). Psychotherapist self-disclosure and transparency in the Internet age. Prof Psychol-Res Pr.

[53] Barak A (2007). Emotional support and suicide prevention through the Internet: a field project report. Comput Hum Behav.

[54] Paxling B, Lundgren S, Norman A, Almlöv J, Carlbring P, Cuijpers P, Andersson G (2013). Therapist behaviours in internet-delivered cognitive behaviour therapy: analyses of e-mail correspondence in the treatment of generalized anxiety disorder. Behav Cogn Psychother.

[55] Cavanagh K, Bennett-Levy J, Richards D, Farrand P (2010). Turn on, tune in and (don’t) drop out: engagement, adherence, attrition, and alliance with internet-based interventions. Oxford Guide to Low Intensity CBT Interventions.

[56] Anderson RE, Spence SH, Donovan CL, March S, Prosser S, Kenardy J (2012). Working alliance in online cognitive behavior therapy for anxiety disorders in youth: comparison with clinic delivery and its role in predicting outcome. J Med Internet Res.

[57] Andersson G, Paxling B, Wiwe M, Vernmark K, Felix CB, Lundborg L, Furmark T, Cuijpers P, Carlbring P (2012). Therapeutic alliance in guided internet-delivered cognitive behavioural treatment of depression, generalized anxiety disorder and social anxiety disorder. Behav Res Ther.

[58] Kelders SM, Bohlmeijer ET, Van Gemert-Pijnen JE (2013). Participants, usage, and use patterns of a web-based intervention for the prevention of depression within a randomized controlled trial. J Med Internet Res.

[59] Johansson O, Michel T, Andersson G, Paxling B (2015). Experiences of non-adherence to Internet-delivered cognitive behavior therapy: a qualitative study. Internet Interv.

[60] Van Gemert-Pijnen JE, Kelders SM, Bohlmeijer ET (2014). Understanding the usage of content in a mental health intervention for depression: an analysis of log data. J Med Internet Res.

[61] Johansson R, Sjöberg E, Sjögren M, Johnsson E, Carlbring P, Andersson T, Rousseau A, Andersson G (2012). Tailored vs. standardized internet-based cognitive behavior therapy for depression and comorbid symptoms: a randomized controlled trial. PLoS One.

