# Supplementary material for: Motivation and Treatment Credibility Predicts Dropout, Treatment Adherence, and Clinical Outcomes in an Internet-Based Cognitive Behavioral Relaxation Program: A Randomized Controlled Trial
Source: J Med Internet Res. 2016 Mar 8;18(3):e52. doi: 10.2196/jmir.5352 (PMC4804106; doi:10.2196/jmir.5352)
Supplement: Multimedia Appendix 1 [file jmir_v18i3e52_app1.pdf]

Multimedia Appendix 1.

Identified candidate predictor variables from the bivariate analyses for each outcome variable.

| | B (SE) | $\chi^2$ | P | OR (95%CI) |
|---|---|---|---|---|
| Early drop out | | | | |
| University education | -0.66 (0.29) | 5.14 | .023 | 0.52 (0.29-0.91) |
| Unemployed or on sick leave | 0.78 (0.46) | 2.92 | .087 | 2.18 (0.89-5.31) |
| ZTPI Future | -0.09 (0.04) | 5.97 | .015 | 0.91 (0.85-0.98) |
| ZTPI Hedonistic | 0.11 (0.05) | 4.11 | .043 | 1.11 (1.01-1.23) |
| ZTPI Fatalistic | 0.08 (0.04) | 4.27 | .039 | 1.08 (1.01-1.16) |
| TCS | -0.14 (0.04) | 10.61 | .001 | 0.87 (0.80-0.95) |
| Attrition during intervention | | | | |
| Baseline stress symptoms | 0.046 (0.02) | 4.21 | .040 | 1.05 (1.01-1.09) |
| ZTPI Future | -0.07 (0.03) | 4.62 | .032 | 0.94 (0.88-0.99) |
| ZTPI Hedonistic | 0.15 (0.05) | 9.92 | .002 | 1.16 (1.06-1.28) |
| ZTPI Fatalistic | 0.09 (0.03) | 8.49 | .004 | 1.10 (1.03-1.18) |
| WAI Total | -0.06 (0.03) | 4.05 | .044 | 0.94 (0.88-0.99) |
| IMI | -0.05 (0.02) | 5.02 | .025 | 0.95 (0.92-0.99) |
| Reliably improved | | | | |
| Baseline stress symptoms | 0.07 (0.03) | 4.48 | .03 | 1.07 (1.01-1.14) |
| TCS | 0.13 (0.04) | 9.48 | .002 | 1.14 (1.05-1.24) |
| WAI Total | 0.19 (0.05) | 12.02 | .001 | 1.21 (1.09-1.34) |
| IMI | 0.08 (0.03) | 8.28 | .004 | 1.08 (1.03-1.15) |

Note. ZTPI = Zimbardo Time Perspective Inventory, TCS = treatment Credibility Scale, WAI = Working Alliance Inventory, IMI = Intrinsic Motivation Inventory.
