# Supplementary material for: Motivation and Treatment Credibility Predicts Dropout, Treatment Adherence, and Clinical Outcomes in an Internet-Based Cognitive Behavioral Relaxation Program: A Randomized Controlled Trial
Source: J Med Internet Res. 2016 Mar 8;18(3):e52. doi: 10.2196/jmir.5352 (PMC4804106; doi:10.2196/jmir.5352)
Supplement: Multimedia Appendix 2 [file jmir_v18i3e52_app2.pdf]

Multimedia Appendix 2.

Identified candidate predictor variables from the bivariate analyses for each outcome variable.

	B (SE)	β	t	P
Treatment progress				
Enhanced support	2.83 (1.53)	0.15	1.85	.067
University education	3.11 (1.24)	0.20	2.51	.013
ZTPI Future	0.40 (0.14)	0.22	2.84	.005
ZTPI Hedonistic	-0.52 (0.21)	-0.20	2.55	.012
TCS	0.32 (0.06)	0.54	5.62	<.001
WAI Total	0.32 (0.09)	0.36	3.67	<.001
IMI	0.23 (0.05)	0.40	4.28	<.001
Registered exercises				
ZTPI Hedonistic	-0.19 (0.10)	-0.17	1.89	.062
TCS	0.08 (0.04)	0.24	2.05	.044
WAI Total	0.11 (0.05)	0.23	2.23	.028
IMI	0.12 (0.03)	0.36	3.70	<.001
Post measurement stress symptoms				
Unemployed or on sick leave	7.38 (2.77)	0.29	2.66	.009
Baseline stress symptoms	0.52 (0.12)	0.44	4.28	<.001
TSRQ EM	0.85 (0.19)	0.44	4.41	<.001
ZTPI Future	-0.50 (0.20)	-0.28	2.58	.012
TCS	-0.31 (0.08)	-0.39	3.69	<.001
IMI	-0.22 (0.10)	-0.27	2.31	.024

Note. ZTPI = Zimbardo Time Perspective Inventory, TCS = treatment Credibility Scale, WAI = Working Alliance Inventory, IMI = Intrinsic Motivation Inventory, TSRQ = Treatment Self-regulation Questionnaire External Motivation.
